# A Phase Ib/II study of IGF-neutralising antibody xentuzumab with enzalutamide in metastatic castration-resistant prostate cancer

**DOI:** 10.1038/s41416-023-02380-1

**Published:** 2023-08-03

**Authors:** Valentine M. Macaulay, Simon Lord, Syed Hussain, José Pablo Maroto, Robert Hugh Jones, Miguel Ángel Climent, Natalie Cook, Chia-Chi Lin, Shian-Shiang Wang, Diletta Bianchini, Mark Bailey, Laura Schlieker, Thomas Bogenrieder, Johann de Bono

**Affiliations:** 1 Nuffield Department of Surgical Sciences, University of Oxford, Oxford, UK; 2 Department of Oncology, University of Oxford, Oxford, UK; 3 Department of Oncology and Metabolism, University of Sheffield, Sheffield, UK; 4 Hospital de la Santa Creu i Sant Pau, Barcelona, Spain; 5 Velindre Cancer Centre and Cardiff University, Cardiff, UK; 6 Instituto Valenciano de Oncología (IVO), Valencia, Spain; 7 The Christie NHS Foundation Trust and the University of Manchester, Manchester, UK; 8 National Taiwan University Hospital, Taipei, Taiwan; 9 Taichung Veterans General Hospital, Taichung, Taiwan; 10 The Institute of Cancer Research, London, UK; 11 The Royal Marsden NHS Foundation Trust, Sutton, London, UK; 12 Boehringer Ingelheim Ltd, Bracknell, Berkshire, UK; 13 External Statistician on behalf of Boehringer Ingelheim GmbH & Co. KG, Staburo GmbH & Co. KG, Munich, Germany; 14 Boehringer Ingelheim RCV GmbH & Co KG, Vienna, Austria; 15 Department of Experimental and Clinical Pharmacology and Pharmacogenomics, University Hospital Tübingen, Tübingen, Germany

**Keywords:** Prostate cancer, Drug discovery, Prostate cancer

## Abstract

**Background:**

This multicentre, open-label, Phase Ib/II trial evaluated the insulin-like growth factor (IGF) 1/2 neutralising antibody xentuzumab plus enzalutamide in metastatic castrate-resistant prostate cancer (mCRPC).

**Methods:**

The trial included Phase Ib escalation and expansion parts and a randomised Phase II part versus enzalutamide alone. Primary endpoints in the Phase Ib escalation, Phase Ib expansion and Phase II parts were maximum tolerated dose (MTD), prostate-specific antigen response and investigator-assessed progression-free survival (PFS), respectively. Patients in the Phase Ib escalation and Phase II parts had progressed on/after docetaxel/abiraterone.

**Results:**

In the Phase Ib escalation (*n* = 10), no dose-limiting toxicities were reported, and xentuzumab 1000 mg weekly plus enzalutamide 160 mg daily (Xe1000 + En160) was defined as the MTD and recommended Phase 2 dose. In the Phase Ib expansion (*n* = 24), median PFS was 8.2 months, and one patient had a confirmed, long-term response. In Phase II (*n* = 86), median PFS for the Xe1000 + En160 and En160 arms was 7.4 and 6.2 months, respectively. Subgroup analysis suggested trends towards benefit with Xe1000 + En160 in patients whose tumours had high levels of *IGF1* mRNA or PTEN protein. Overall, the combination was well tolerated.

**Conclusions:**

Xentuzumab plus enzalutamide was tolerable but lacked antitumour activity in unselected patients with mCRPC.

**Clinical trial registration:**

EudraCT number 2013-004011-41.

## Introduction

Androgen deprivation therapy is standard of care for advanced or metastatic prostate cancer [1]. Unfortunately, most patients will progress to a castration-resistant disease state after 2–3 years [2, 3]. Recognition that androgen receptor (AR) signalling is a key driver of castration-resistant prostate cancer (CRPC) [2] led to the development of potent inhibitors of the AR pathway. Enzalutamide, a second-generation androgen antagonist, targets multiple steps in the AR signalling pathway and is licensed for use in CRPC [4, 5]. Nevertheless, most patients eventually develop resistance [6], highlighting a need for novel agents for patients with refractory disease.

The insulin-like growth factor (IGF) axis plays an important role in prostate cancer progression. Castration leads to increased signalling via the type I IGF tyrosine kinase receptor (IGF-1R), activating the PI3K/AKT/mTOR pathway [7] which may lead to androgen-independent AR transactivation, facilitating progression to castration-resistance [8]. IGF-1R activation and signalling has also been shown to dephosphorylate AR, enhancing AR translocation to the nucleus [9]. Thus, there is biological rationale for co-targeting AR and IGF signalling in patients with CRPC.

Xentuzumab is a humanised monoclonal antibody that binds to and neutralises the IGF-1 and IGF-2 ligands [10]. Xentuzumab has demonstrated antitumour activity in preclinical studies [10], including in combination with enzalutamide in prostate cancer models [11]. In Phase I trials undertaken in patients with advanced solid tumours, xentuzumab monotherapy has demonstrated manageable tolerability and antitumour activity [12].

This Phase Ib/II trial evaluated xentuzumab plus enzalutamide in patients with metastatic CRPC (mCRPC). In patients who had progressed on docetaxel-based chemotherapy and abiraterone, a Phase Ib dose escalation part was conducted to determine the maximum tolerated dose (MTD), and a randomised Phase II part assessed the combination versus enzalutamide alone. A Phase Ib expansion cohort evaluated the addition of xentuzumab to enzalutamide in docetaxel/abiraterone naïve patients experiencing prostate-specific antigen (PSA) progression on enzalutamide.

## Methods

### Study design and patients

This multicentre, open-label, Phase Ib/II trial was conducted in 27 centres in eight countries. The trial comprised: a Phase Ib 3 + 3 escalation phase to determine the MTD and/or recommended Phase II dose (RP2D) of xentuzumab plus enzalutamide in patients with mCRPC after docetaxel and abiraterone; a Phase Ib exploratory expansion phase in patients with mCRPC and PSA progression on enzalutamide; and a Phase II phase, where patients with mCRPC were randomised (1:1) to xentuzumab plus enzalutamide or enzalutamide alone. Randomisation was performed centrally via an Interactive Voice/Web Response system.

Eligible patients were adults with histologically or cytologically confirmed adenocarcinoma of the prostate and radiographic evidence of metastatic disease (stage M1 or D2). Other eligibility criteria were: PSA ≥20 ng/ml (later amended to ≥5 ng/ml) and prior surgical or chemical castration with a serum testosterone of <50 ng/ml; Eastern Cooperative Oncology Group performance status (ECOG PS) of 0 or 1 (ECOG PS 2 was permitted in the dose escalation part); and adequate organ function.

For the Phase Ib escalation and Phase II parts, patients had experienced progressive disease (PD) while, or after, receiving docetaxel and abiraterone. They were required to have received ≥12 weeks of docetaxel and were intolerant to, or unlikely to derive benefit from, further docetaxel-based therapy. PD was defined as progressive measurable disease per Response Evaluation Criteria in Solid Tumors (RECIST) v1.1, bone scan progression (at least two new lesions plus rising PSA values), or an increasing PSA level (at least two consecutive rising values taken at least 1 week apart). Patients must not have received more than two prior non-docetaxel-containing cytotoxic chemotherapy regimens for mCRPC, a taxane-based treatment or abiraterone within 4 weeks prior to study treatment start, or prior enzalutamide in any setting. For the exploratory expansion cohort, patients had to be receiving enzalutamide and had PSA progression with at least two consecutive rising values at least 1 week apart. Patients must not have received prior taxane-based chemotherapy or abiraterone in any setting.

Other exclusion criteria were prior therapy with agents targeting the IGF pathway; prior chemotherapy, immunotherapy, biological therapies, molecular targeted therapy, hormone therapy (except luteinizing hormone-releasing hormone agonists/antagonists), radiotherapy (except localised therapy for analgesic purposes or for lytic lesions at risk of fracture) within 4 weeks of starting trial treatment; small cell or neuroendocrine tumours; known or suspected leptomeningeal metastases; uncontrolled or poorly controlled hypertension; previous or concomitant malignancies at any other site (except for benign basal cell carcinoma, benign low grade transitional cell carcinoma of the bladder, or an effectively treated malignancy that had been in remission for >5 years and was considered cured).

The trial was conducted in accordance with the principles of the Declaration of Helsinki and Good Clinical Practice. The protocol was approved by the independent ethics committees (IECs) and institutional review boards of the participating centres The IEC (National Research Ethics Service Committee London – Chelsea Research Ethics Committee Bristol Centre, Bristol, United Kingdom) of the Coordinating Investigator gave a favourable opinion for the study on 14 August 2014. All patients provided written informed consent.

### Treatment

In the Phase Ib escalation, patients received xentuzumab at a starting dose of 750 mg weekly (intravenous infusion) plus enzalutamide 160 mg daily (oral; Xe750 + En160), escalating to xentuzumab 1000 mg weekly plus enzalutamide 160 mg daily (Xe1000 + En160) (Supplementary Fig. 1). In the Phase Ib expansion, patients received xentuzumab plus enzalutamide at the MTD/RP2D. In the Phase II part, patients were randomised to xentuzumab plus enzalutamide (MTD/RP2D) or enzalutamide alone (160 mg daily). Treatment continued until disease progression, intolerable adverse events (AEs) or other reasons for withdrawal.

### Endpoints and assessments

In the Phase Ib escalation, the primary endpoint was the MTD of xentuzumab based on occurrence of dose-limiting toxicities (DLTs). MTD was defined as the dose level at which ≤1 DLT was observed in six patients during the first 28-day cycle of treatment. DLTs were defined as per the criteria in the Supplementary Methods.

In the Phase Ib expansion, the primary endpoint was PSA response (defined as a decline in PSA value >50% compared with baseline and confirmed by the next available value at least 3 weeks later). Secondary endpoints were investigator-assessed progression-free survival (PFS) and circulating tumour cell (CTC) response. PFS was defined as time from randomisation until radiological tumour progression in bone (based on Prostate Cancer Clinical Trials Working Group 2 [PCWG2] criteria) or soft tissue (based on modified RECIST v1.1), whichever occurred earlier, or death from any cause. CTC response was defined as a CTC reduction from ≥5 to <5 cells per 7.5 ml blood for at least one post-baseline timepoint

The primary endpoint in Phase II was investigator-assessed PFS. Secondary endpoints were centrally-assessed PFS, overall survival (OS; defined as the time from randomisation to death from any cause), time to PSA progression (defined as time from first treatment until a ≥25% increase in PSA and an absolute increase of >2 ng/ml from the nadir, in cases where there had been a PSA decline from baseline before increasing, or from the baseline if there had been no PSA decline from baseline), maximum decline in PSA, percentage change in PSA at Week 12, PSA response (as defined above) and CTC response assessed by three criteria: CTC reduction (decline from ≥5 to <5 cells per 7.5 ml blood for at least one post-baseline timepoint), maximum decline in CTC counts from baseline, and CTC status at Week 12 (≥5 to <5 cells per 7.5 ml blood).

Tumours were assessed at screening by computed tomography or magnetic resonance imaging of the chest, abdomen and pelvis, and a bone scan. The same radiographic procedure had to be used throughout the study. In the Phase Ib escalation, imaging was performed every 12 weeks. In the Phase Ib expansion and Phase II parts, imaging was performed at baseline, every 2 cycles (8 weeks) up to Week 24 and every 3 cycles (12 weeks) thereafter. All imaging data collected in the Phase II part were sent to a central imaging unit to obtain an independent blinded confirmation of tumour response assessment.

PSA assessments were performed locally. Blood samples were taken at screening, baseline, at the start of cycle 3, and every cycle thereafter. Upon PSA progression, patients were to be kept on trial treatment until radiological or symptomatic progression was documented. Blood samples for CTC assessment were taken at baseline, at the beginning of cycles 1–3, 5 and 7, and every 12 weeks thereafter. CTCs were analysed by cell counting at an authorised Contract Research Organisation (Veridex CellSearch technology).

Safety was assessed by incidence and severity of AEs which were graded according to Common Terminology Criteria for Adverse Events version 4.03. The Brief Pain Inventory short form was used in all parts of the trial to assess pain at the start of each cycle [13]. The Functional Assessment of Cancer Therapy-Prostate questionnaire was used to assess quality of life in the Phase II part, at the start of cycles 1, 2, 3, 5, 7 and 10, and every 12 weeks thereafter [14].

Evaluation of biomarkers was conducted as an exploratory objective of the trial. Blood samples were taken for quantification of free and total IGF-1 and IGF-2, total IGF binding protein (IGFBP)-3 and IGF bioactivity. IGF-1, IGF-2 and IGFBP-3 were quantified using validated immunoassays. IGF bioactivity was analysed in plasma by the quantification of IGF-1R-phosphorylation in cells expressing human IGF-1R. PTEN expression was assessed by immunohistochemistry (IHC) in archival formalin-fixed, paraffin-embedded (FFPE) tumour samples and was undertaken centrally by Discovery Life Sciences (Kassel, Germany) using the antibody clone 138G6 (cat #9559, Cell Signaling Technology) and the fully automated Ventana Benchmark Ultra platform following the Ultra View DAB procedure (cat #760-500, Ventana Medical Systems/Roche Diagnostics).

RNA and DNA were extracted from archival FFPE tumour tissue, to determine gene expression profiles and mutational status (FoundationOne Assay, bait set T7; Foundation Medicine, Cambridge, MA [15]) respectively. Blood samples were collected at the start of cycles 1, 3, and 4 and after disease progression. DNA was extracted for genotyping of *IGFBP3* gene polymorphisms and circulating free DNA (cfDNA) was extracted from plasma for mutation analysis using a predesigned gene panel from Qiagen.

### Statistical analysis

In the Phase Ib escalation, 9–12 patients were planned to be assessed, assuming that two cohorts were needed to determine the MTD, 3–6 patients per cohort. In the Phase Ib expansion, a 30% PSA response rate was assumed [13], and therefore 25 patients were planned to be treated to obtain a sample size of 21 evaluable patients for a probability of ~80% of observing at least five patients with a PSA response. Sample size for Phase II was calculated based on a hazard ratio for PFS of 0.65 for xentuzumab plus enzalutamide versus enzalutamide alone. A sample size of 80 patients (40 per arm) was planned.

PFS and OS were assessed based on the Kaplan–Meier method. Point estimates, together with confidence intervals (CIs; based on Greenwood’s method) were provided for median time to event and quartiles. An estimation of the effect of xentuzumab plus enzalutamide on PFS compared with enzalutamide alone was given by the hazard ratio (HR) and its 95% CIs using a Cox proportional hazards model (HR <1 favoured treatment with xentuzumab plus enzalutamide) and an exploratory *p* value from the two-sided log rank test was calculated. The consistency of treatment effect on PFS across subgroups was investigated in the Phase II part; the cut-off for continuous biomarkers was determined using a model optimising the partial likelihood with regard to PFS based on investigator assessment. Analyses of AEs were descriptive.

## Results

### Phase Ib escalation

#### Patients and treatment

**Fig. 1:**
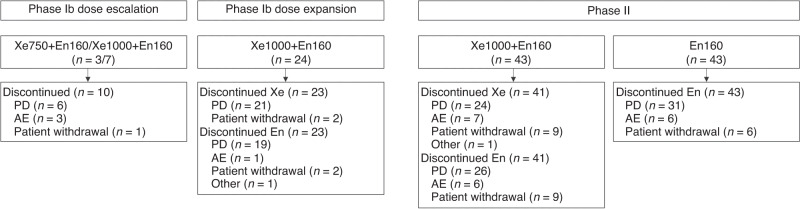
Patient disposition. *AE* adverse event, *En160* enzalutamide 160 mg daily, *PD* progressive disease, *Xe750* + *En160* xentuzumab 750 mg weekly plus enzalutamide 160 mg daily, *Xe1000* + *En160* xentuzumab 1000 mg weekly plus enzalutamide 160 mg daily.

The Phase Ib escalation part was conducted at four sites in the UK. Ten patients were treated (Table 1); three received Xe750 + En160 and seven received Xe1000 + En160. All patients had discontinued treatment at data cut-off (October 2019; Fig. 1). Median duration of treatment was 2.6 months (range 0.9–10.8).

**Table 1:** Patient demographics.

	Phase I escalation	Phase I expansion	Randomised Phase II	
	Xe750/1000 + En160 (*n* = 10)	Xe1000 + En160 (*n* = 24)	Xe1000 + En160 (*n* = 43)	En160 (*n* = 43)
Median age, years (range)	71.5 (55–79)	74.0 (55–89)	68.0 (46–88)	72.0 (51–82)
Race, *n* (%)				
Asian	0	0	15 (34.9)	10 (23.3)
White	10 (100)	23 (95.8)	27 (62.8)	33 (76.7)
Black/African American	0	1 (4.2)	0	0
Missing	0	0	1 (2.3)	0
ECOG PS, *n* (%)				
0	3 (30.0)	9 (37.5)	14 (32.6)	21 (48.8)
1	6 (60.0)	15 (62.5)	29 (67.4)	22 (51.2)
2	1 (10.0)	0	0	0
Smoking status, *n* (%)				
Never smoked	3 (30.0)	13 (54.2)	22 (51.2)	26 (60.5)
Ex-smoker	6 (60.0)	11 (45.8)	16 (37.2)	14 (32.6)
Current smoker	1 (10.0)	0	5 (11.6)	3 (7.0)
Median time since first diagnosis, months (range)	81.5 (26.9–245.2)	56.1 (15.4–198.5)	69.8 (6.3–200.9)	68.5 (21.6–239.8)
Visceral involvement at screening, *n* (%)	4 (40.0)	0	8 (18.6)	11 (25.6)
Bone involvement at screening, *n* (%)	10 (100)	19 (79.2)	43 (100)	40 (93.0)
Gleason total score, *n* (%)				
2–6	1 (10.0)	1 (4.2)	1 (2.3)	3 (7.0)
7	3 (30.0)	4 (16.7)	7 (16.3)	15 (34.9)
8	2 (20.0)	3 (12.5)	10 (23.3)	7 (16.3)
9	2 (20.0)	14 (58.3)	19 (44.2)	15 (34.9)
10	0	0	2 (4.7)	2 (4.7)
Missing	2 (20.0)	2 (8.3)	4 (9.3)	1 (2.3)
Median PSA, µg/l (range)	286.1 (60.0–1087.0)	36.0 (6.7–1232.7)	217.6 (6.8–3616.7)	147.9 (7.5–9106.0)

*ECOG PS* Eastern Cooperative Oncology Group performance status, *PSA* prostate-specific antigen, *Xe1000* + *En160* xentuzumab 1000 mg weekly plus enzalutamide 160 mg daily, *Xe750* + *En160* xentuzumab 750 mg weekly plus enzalutamide 160 mg daily.

#### MTD determination

Nine patients were evaluable for MTD (one was not evaluable due to missing >3 consecutive doses of enzalutamide in cycle 1). There were no DLTs in either dose cohort in cycle 1. Based on the definition of the MTD as the dose level at which ≤1 DLT was observed in six patients during cycle 1, Xe1000 + En160 was defined as the MTD and the RP2D.

#### Safety

All patients reported a treatment-emergent AE (including two grade 4 events [spinal cord compression and condition aggravated] and one grade 5 event [malignant neoplasm progression]). None of the grade 4 or 5 AEs were considered drug-related. The most frequent drug-related AEs were fatigue (70.0%) and decreased appetite (50.0%; Supplementary Table 1). There was one serious AE (SAE) that was considered drug related: a grade 1 infusion-related reaction. Three patients in the Xe1000 + En160 cohort had AEs leading to discontinuation of xentuzumab and enzalutamide.

### Phase Ib expansion

#### Patients and treatment

The Phase Ib expansion was conducted at seven sites in Spain, the UK and the USA. Twenty-four patients were treated (Table 1), and one patient remained on treatment at data cut-off (Fig. 1). Median duration of treatment was 4.1 months (range 0.3–35.0); median duration of xentuzumab was slightly shorter than overall treatment duration (median [range] 3.6 months [0.3–34.7]).

#### Efficacy

**Fig. 2:**
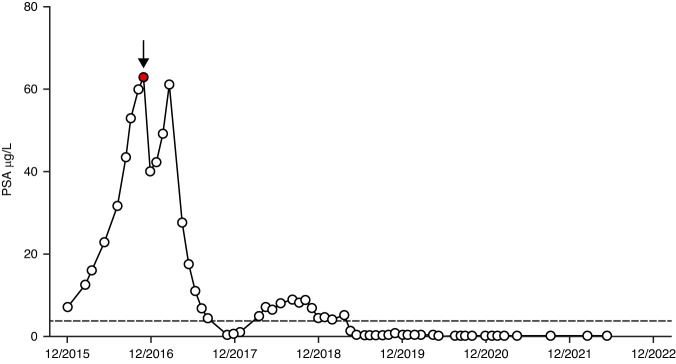
PSA profile of patient with extended response to Xen1000 + En160. The patient presented at age 64 in 2011 with PSA 83, bilateral prostate adenocarcinoma Gleason grade group 5 (4 + 5 = 9) and left acetabular metastasis on MRI scan. He was treated with leuprorelin, enzalutamide 160 mg daily was added in May 2015 but after initial PSA response there was PSA progression. CT scan May 2016 showed pelvic nodal progression (left pelvic side-wall node 19 × 15 mm, right external iliac and common iliac nodes, largest 16 × 14 mm). In November 2016, he was recruited to the Phase Ib expansion part of the trial. Baseline assessment showed ECOG PS 0, PSA 62.98, with increase in retroperitoneal and pelvic lymphadenopathy since May 2016. He remained on enzalutamide and weekly xentuzumab was added. Enzalutamide was dose reduced (120 mg daily) after he developed grade 1 QTc prolongation, intermittent atrial fibrillation and hypertension requiring adjustment of antihypertensive medication and anticoagulation (edoxaban 60 mg daily, replaced by aspirin 75 mg April 2021 after reversion to sinus rhythm). Xentuzumab infusions were interrupted April–August 2020 (due to COVID-related restricted access to the Trials Unit) and subsequently re-started. Trial treatment continues, scans showing pelvic nodes below size criteria with no evidence of bone metastases. Graph: PSA values prior to and on trial. Arrow: baseline PSA at trial entry. Dotted line: upper limit of normal. *ECOG PS* Eastern Cooperative Oncology Group performance status, *PSA* prostate-specific antigen.

One patient (4.2%) in the expansion cohort had a confirmed PSA response; the patient had a long-term PSA and scan response and remained on study at data cut-off (Fig. 2). Limited biomarker evaluation of this patient’s archival FFPE diagnostic biopsy showed a PTEN H-score of 100. A further patient had an unconfirmed PSA response. Fifteen patients (62.5%) in the expansion cohort had experienced a PFS event at data cut-off; median PFS was 8.2 months (95% CI 3.5–14.6). Nine patients had ≥5 CTC per 7.5 ml blood at baseline; of these, one patient (11.1%) had a CTC response.

#### Safety

All patients reported a treatment-emergent AE. No grade 4 or 5 AEs were reported. Drug-related AEs were reported in 21 (87.5%) patients; most frequently, fatigue (25.0%) and decreased appetite (20.8%; Supplementary Table 2). Two patients had drug-related SAEs (grade 2 atrial fibrillation and grade 1 electrocardiogram T-wave inversion), and three and five patients, respectively, discontinued xentuzumab and enzalutamide due to AEs.

### Randomised Phase II

#### Patients and treatment

This phase was conducted at 23 sites in seven countries (Hong Kong, Netherlands, Singapore, South Korea, Spain, Taiwan and the UK). Eighty-six patients were randomised to Xe1000 + En160 (*n* = 43) and En160 (*n* = 43). Patient demographics are shown in Table 1. The treatment arms were imbalanced with respect to ECOG PS (more patients in the Xe1000 + En160 arm had a score of 1) and Gleason score (more patients in the Xe1000 + En160 arm had a score of ≥8). Two patients remained on treatment at data cut-off, both in the Xe1000 + En160 cohort; Fig. 1). Median duration (range) of treatment was similar in both arms (Xe1000 + En160: 3.2 [0.5–40.1] months vs En160: 3.7 [0.5–32.2] months).

#### Efficacy

**Fig. 3:**
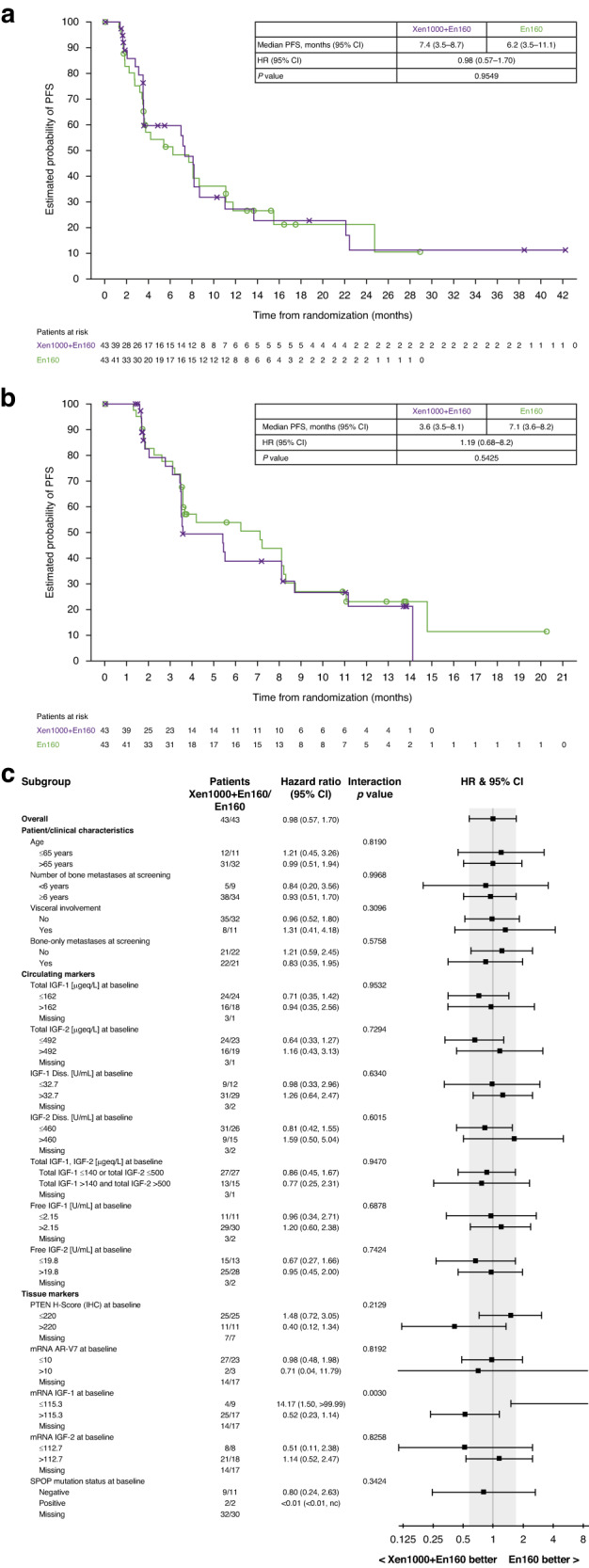
Progression-free survival for xentuzumab plus enzalutamide versus enzalutamide alone in the randomised Phase II part of the study. **a** According to investigator assessment. **b** According to central review. **c** Subgroup analysis of progression-free survival by investigator assessment.

At data cut-off, 24 patients (55.8%) in the Xe1000 + En160 arm had experienced a PFS event versus 29 patients (67.4%) in the En160 arm. Investigator-assessed median PFS was 7.4 months in the Xe1000 + En160 arm and 6.2 months in the En160 arm (HR 0.98, [95% CI 0.57–1.70]; Fig. 3a). Results were similar when adjusted in a post-hoc analysis for baseline imbalances between treatment arms in ECOG PS and Gleason score (HR 0.83 [95% CI 0.47–1.46]).

**Fig. 4:**
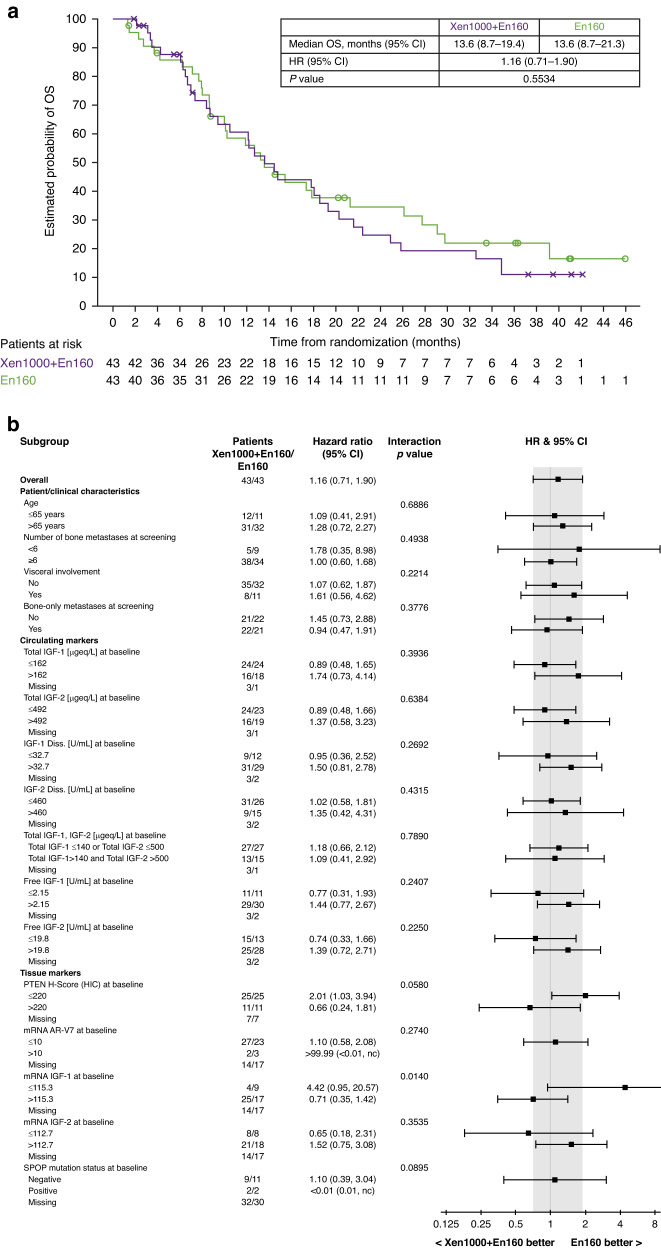
Overall survival for xentuzumab plus enzalutamide versus enzalutamide alone in the randomised Phase II part of the study. **a** Overall survival analyses. **b** Subgroup overall survival analyses.

There was no evidence of a difference in PFS between treatment arms by central review. Median PFS was 3.6 months in the Xe1000 + En160 arm versus 7.1 months in the En160 arm (HR 1.19 [95% CI 0.68–2.06]; Fig. 3b). Subgroup analyses based on patient and clinical parameters, circulating markers and tissue markers were generally consistent with the primary results (Fig. 3c). At data cut-off, 64 patients had died (33 in the Xe1000 + En160 arm and 31 in the En160 arm); no difference in OS was observed between the treatment arms (median 13.6 months in each arm; HR 1.16 [95% CI 0.71–1.90]; Fig. 4a). Again, subgroup analyses were generally consistent with primary results (Fig. 4b).

PSA response rates and changes in PSA levels were similar in both arms (Table 2). Median time to PSA progression was 4.6 months in the Xe1000 + En160 arm and 3.7 months in the En160 arm (HR 0.64 [95% CI 0.35–1.18]). CTC results are shown in Table 2; there were no notable differences in CTC response between treatment arms. There were no differences between treatment arms with respect to pain worsening, pain palliation or quality of life (data not shown).

**Table 2:** PSA-related and CTC endpoints in the randomised Phase II part.

	Xe1000 + En160 (*n* = 43)	En160 (*n* = 43)
PSA response, *n* (%)		
Yes	9 (20.9)	8 (18.6)
Confirmed	7 (16.3)	8 (18.6)
Unconfirmed	2 (4.7)	0
No	31 (72.1)	29 (67.4)
Missing	3 (7.0)	6 (14.0)
Maximum decline in PSA compared to baseline, µg/l		
* N*	40	37
Median (range)	–20.4 (–2803.8, 1210.2)	–9.0 (–5857.0, 1646.2)
Maximum decline in PSA compared to baseline, %		
* N*	40	37
Median (range)	–8.5 (–99.8, 272.7)	–8.1 (–97.5, 287.9)
Change in PSA from baseline to week 12, %		
* N*	31	30
Median (range)	18.6 (–99.2, 251.1)	18.3 (–94.8, 360.7)
CTC status at week 12, *n* (%)		
≥5 cells per 7.5 ml blood	26 (60.5)	20 (46.5)
<5 cells per 7.5 ml blood	11 (25.6)	16 (37.2)
Missing on-treatment value	6 (14.0)	7 (16.3)
Odds ratio (95% CI); *p* value	1.89 (0.73, 5.06); 0.192	
Maximum decline in CTC counts, %		
* N*	32	28
Median (range)	–46.3 (–100.0, 1153.4)	–34.5 (–100.0, 933.3)
CTC response, *n* (%)		
*N* (patients with baseline CTC value ≥5 cells per 7.5 ml)	25	19
Yes	4 (16.0)	2 (10.5)
No	19 (76.0)	15 (78.9)
Missing on-treatment value	2 (8.0)	2 (10.5)
Odds ratio (95% CI); *p* value	1.58 (0.27, 12.52); 0.619	

*CI* confidence interval, *CTC* circulating tumour cell, *PSA* prostate-specific antigen, *Xe1000* + *En160* xentuzumab 1000 mg weekly plus enzalutamide 160 mg daily.

#### Biomarkers and pharmacogenomic evaluation

Circulating and tissue markers were assessed for prognostic and predictive potential. In the Xe1000 + En160 arm, total circulating IGF-1 and IGF-2 concentrations increased after the start of xentuzumab treatment, reaching a plateau by the start of cycle 2; free IGF-1 and bioactive IGF decreased following xentuzumab treatment. While circulating biomarkers showed prognostic potential, e.g., high levels of IGF-1 and IGF-2 at baseline tended to be associated with better PFS and OS regardless of treatment (data not shown), none of the circulating biomarkers tested showed predictive potential for PFS or OS (Figs. 3c and 4b).

Archival prostate tissue samples were analysed for PTEN expression by IHC in 90 patients, including 72 Phase II patients (36 per arm) and 18 patients from the Phase 1b expansion part. Median PTEN H-score was 177.5 in the Xe1000 + En160 arm, 100.0 in the En160 arm and 130 in all 90 patients. PTEN H-score was not prognostic for PFS or OS (data not shown). There was no OS difference in the Xe1000 + En160 versus En160 arms in patients whose tumours had PTEN H-score >80 (17.8 vs 17.4 months; HR 1.07, 95% CI 0.52–2.20) or >130 (17.8 vs 17.4 months; HR 1.18, 95% CI 0.55–2.53). However, there was a trend towards OS benefit with Xe1000 + En160 versus En160 in patients with tumours with PTEN H-score >220 (median OS 19.4 vs 12.7 months; HR 0.66 [95% CI 0.24–1.81]; Fig. 4b). In contrast, in patients with PTEN H-score of ≤220, OS was longer in the En160 arm (median OS: 10.5 vs 21.3 months; HR 2.01 [95% CI 1.03–3.94]; Fig. 4b).

Expression of IGF-pathway-related genes was determined in archival tissue samples from 55 patients (Xe1000 + En160, 29 patients; En160, 26 patients). Low expression of *CDK6, EGFR, ETS1, MAPK3, PIK3CA, RPS6KB1* or *SOS1* showed prognostic potential for longer PFS, and low expression of *BCL2, CDK6, EGFR, ESR1, ETS1, IGF2a, MAPK1, MPAK3, PIK3CA* and *SOS1* was prognostic for longer OS (data not shown). The analysed genes were also assessed for their predictive potential for PFS and OS. Of note, high *IGF1* mRNA levels (above 115.3) appeared to be associated with trends towards improved PFS (HR 0.52 [95% CI 0.23–1.14]; interaction *p* = 0.003) and OS (HR 0.71 [95% CI 0.35–1.42]; interaction *p* = 0.014) in the Xe1000 + En160 arm, although this was based on small sample sizes (Figs. 2c and 3b). In addition, trends towards PFS benefit with Xe1000 + En160 were observed if expression of *CCND1, CDKN1B, CDKN1C, ELK1, IGF1R, IGFBP2, IGFBP5, MAPK3*, or *SOS1* was high or if expression of *AR b, BRAF, CCND3, IGFBP3*, or *INSR* was low (interaction p-values were <0.1). Trends towards OS benefit with Xe1000 + En160 were observed if expression of *CCND1, CDKN1B, CDKN1C, IGFBP2, IRS1, MAPK1*, or *SOS1* was high or if expression of *AR b, BRAF, ERG, IGFBP3, INSR*, or *PGRa* was low.

Tumour mutations were analysed in archival FFPE samples from 24 patients (Xe1000 + En160, 11 patients; En160, 13 patients). Median number of mutations per tumour was 14 in the Xe1000 + En160 arm and nine in the control arm. Assessment of prognostic potential showed that patients were more likely to stay progression free and alive if their tumour showed ≤10 mutations (>10 vs ≤10 mutations: HR 0.21 [95% CI 0.07–0.70]) or if their tumour had ≤9 short variants (>9 vs ≤9 short variants: HR 0.08 [95% CI 0.02–0.39]). Patients were more likely to stay alive for a longer period of time if their tumour had ≤9 short variants (>9 vs ≤9 short variants; HR 0.135 [95% CI 0.040–0.459]). Mutational status by cfDNA showed that the mean (standard deviation [SD]) number of mutations at baseline was similar in both treatment groups (Xe1000 + En160: 2.2 [1.5], *n* = 25; En160: 1.8 [1.1], *n* = 21). At the end of treatment, the mean (SD) number of mutations was slightly higher in both treatment arms (Xe1000 + En160: 4.1 [5.5], *n* = 22; En160: 3.9 [5.4], *n* = 17). None of the analysed mutations showed prognostic potential for PFS or OS.

#### Safety

All patients reported at least one treatment-emergent AE. Two patients in the Xe1000 + En160 arm had an AE leading to death (respiratory failure and general physical health deterioration); neither were considered related to treatment. Drug-related AEs occurred in 41 (95.3%) of patients in the Xe1000 + En160 arm and 35 (81.4%) in the En160 arm. The most common drug-related AEs are shown in Table 3. Three patients had drug-related SAEs in the Xe1000 + En160 arm (grade 1 vomiting, grade 3 fatigue and grade 4 general physical health deterioration) and one patient in the En160 arm had a drug-related SAE (grade 3 asthenia). Overall, nine patients (20.9%) in Xe1000 + En160 arm discontinued treatment with xentuzumab due to AEs, most commonly due to fatigue (three patients [7.0%]). Seven patients (16.3%) in the Xe1000 + En160 arm and eight (18.6%) in the En160 arm discontinued enzalutamide treatment due to AEs.

**Table 3:** Most common drug-related adverse events reported in the randomised Phase II part (occurring in ≥10% of patients in either treatment arm).

	Xe1000 + En160 (*n* = 43)		En160 (*n* = 43)	
	All grades	Grade ≥3	All grades	Grade ≥3
Any DRAE, *n* (%)	41 (95.3)	16 (37.2)	35 (81.4)	10 (23.3)
Fatigue, *n* (%)	24 (55.8)	8 (18.6)	17 (39.5)	4 (9.3)
Decreased appetite, *n* (%)	20 (46.5)	1 (2.3)	13 (30.2)	0
Nausea, *n* (%)	6 (14.0)	1 (2.3)	9 (20.9)	0
Anaemia, *n* (%)	5 (11.6)	3 (7.0)	5 (11.6)	4 (9.3)
Diarrhoea, *n* (%)	5 (11.6)	0	3 (7.0)	0
Vomiting, *n* (%)	5 (11.6)	0	0	0
Weight decreased, *n* (%)	8 (18.6)	1 (2.3)	2 (4.7)	0
Asthenia, *n* (%)	4 (9.3)	0	7 (16.3)	1 (2.3)
Hot flush, *n* (%)	2 (4.7)	0	6 (14.0)	0

*DRAE* drug-related adverse event, *Xe1000* + *En160* xentuzumab 1000 mg weekly plus enzalutamide 160 mg daily.

## Discussion

Xentuzumab in combination with enzalutamide demonstrated a manageable safety profile across all three parts of the study. No DLTs were reported in the first cycle during the Phase Ib dose escalation phase and the MTD/RP2D was determined to be Xe1000 + En160. The most common AEs across all parts of the study were fatigue and decreased appetite. There were no notable differences in safety profile between the xentuzumab plus enzalutamide and enzalutamide alone arms in the Phase II part. No new safety signals were observed, and the AE profile was as expected based on previous xentuzumab monotherapy trials [12] and the known profile of enzalutamide [5].

The exploratory dose expansion phase suggested that xentuzumab did not have antitumour activity in combination with enzalutamide in patients with mCRPC with rising PSA levels on enzalutamide. Addition of xentuzumab to enzalutamide in the Phase II part did not prolong PFS versus enzalutamide alone in patients with mCRPC after previous treatment with docetaxel and abiraterone. The groups were imbalanced with respect to baseline characteristics, with more adverse factors (ECOG PS, Gleason score, mutation burden) in the Xen1000 + En160 arm, although the outcomes with respect to PFS and OS were not altered after correction for PFS and Gleason score. Thus, while there is a strong preclinical rationale for targeting of IGF in prostate cancer, this did not translate to clinical efficacy in this study. This finding is consistent with other trials of IGF-1R inhibitory drugs in mCRPC patients. In a study of chemotherapy-naïve patients, addition of the IGF-1R inhibitory antibody, figitumumab, to docetaxel/prednisone did not significantly improve the PSA response rate above the null value of 45%, had a detrimental effect on PFS (4.9 vs 7.9 months) and substantially increased toxicity versus docetaxel/prednisone alone [16]. Another IGF-1R antibody, cixutumumab, had limited antitumour activity in combination with the mTORC1 inhibitor temsirolimus, with an unexpectedly high degree of toxicity and no patient having a >50% PSA decrease from baseline [17]. While the IGF-1R/INSR tyrosine kinase inhibitor linsitinib was well tolerated in a study of patients with chemotherapy-naïve mCRPC, there was no evidence of antitumour activity [18].

Although the current and previous studies indicate that IGF or IGF-1R inhibition do not confer clinical benefit in unselected patients, certain patients with mCRPC appear to benefit. Therefore, there is a need to identify predictive biomarkers that might identify patients most likely to respond. As part of the current trial, there was an exploratory analysis of biomarkers to expand understanding of the disease and study treatment. PTEN is frequently downregulated by gene deletion or mutation as prostate cancers progress to mCRPC [19], with evidence that *PTEN* gene loss correlates with reduction or loss of PTEN signal by IHC [20]. We tested PTEN H-score cut-offs of >130 (the median value for all tumours tested) and >80 vs 0–80, the latter reported to reflect heterozygous *PTEN* loss [21] but neither were predictive. There were too few tumours with H-score 0–10 (12/90, 1.3%) to test the predictive power of very low PTEN signal consistent with biallelic loss of *PTEN* [21]. In patients whose tumours expressed high PTEN (H-score >220) there was a trend towards improved OS in those on Xen1000 + En160 versus En160. Conversely there was a trend towards lack of OS benefit in patients with low PTEN tumours. These observations provide initial clinical support for preclinical data suggesting that PTEN status may be a marker of responsiveness to xentuzumab plus enzalutamide. In PTEN proficient prostate cancer cells and xenografts, treatment with xentuzumab plus enzalutamide was growth inhibitory, but treatment resistance was induced upon PTEN depletion [11]. Using the 220 H-score cut-point, the diagnostic biopsy of the exceptional responder contained relatively low PTEN (H-score 100, Fig. 2). However, primary prostate cancers show intra-tumoral heterogeneity that is poorly captured at diagnostic biopsy, and it is increasingly recognised that clones with metastatic potential are identifiable only at the genetic level [22, 23]. Therefore, PTEN IHC on diagnostic biopsies cannot be used reliably to infer PTEN status of metastatic sites, and it would have been preferable to have had access to tumour tissue biopsied at trial entry to ascertain current PTEN status. As a result, we cannot exclude PTEN proficiency as a driver for response to xentuzumab plus enzalutamide.

Several IGF-pathway-related genes, including *IGF1, IGF1R* and *IGFBP5* were identified as potential predictive biomarkers in this study, with high expression associated with PFS benefit from xentuzumab plus enzalutamide. IGFBP5 is a well-characterised transcriptional readout of IGF axis activity in multiple cell types [24–27]. Thus, *IGF1* and *IGFBP5* upregulation reflect high baseline IGF axis activity; it is plausible that this state could indicate IGF-dependence, potentially contributing to possible benefit from xentuzumab. Another potential biomarker of interest identified in this study was *ERG* gene expression. Patients appeared to be more likely to derive OS benefit from xentuzumab plus enzalutamide if tumour expression of *ERG* was low. This observation contrasts with an in vitro study where *ERG* silencing reduced the sensitivity of prostate cancer cells to IGF-1R inhibition [28]. While these observations are of interest, patients in this trial were not preselected or stratified for PTEN status or transcriptional profiles, and further studies would be required to assess these parameters as predictive biomarkers for response to IGF inhibition in CRPC.

In conclusion, while xentuzumab and enzalutamide can be safely combined, the combination did not show antitumour activity in the overall population of patients with mCRPC. Further assessment of potential markers of response or resistance is required to enable selection of patients who may benefit from this combination.

## Supplementary information

Supplementary Material

## Data Availability

To ensure independent interpretation of clinical study results and enable authors to fulfil their role and obligations under the ICMJE criteria, Boehringer Ingelheim grants all external authors access to relevant clinical study data pertinent to the development of the publication. In adherence with the Boehringer Ingelheim Policy on Transparency and Publication of Clinical Study Data, scientific and medical researchers can request access to clinical study data after publication of the primary manuscript in a peer-reviewed journal, regulatory activities are complete and other criteria are met. Researchers should use the https://vivli.org/ link to request access to study data and visit https://www.mystudywindow.com/msw/datasharing for further information.
