# Supplementary material for: A Phase Ib/II study of IGF-neutralising antibody xentuzumab with enzalutamide in metastatic castration-resistant prostate cancer
Source: Br J Cancer. 2023 Aug 3;129(6):965–73. doi: 10.1038/s41416-023-02380-1 (PMC10491782; doi:10.1038/s41416-023-02380-1)
Supplement: Supplementary file 1 — Supplementary Material [file 41416_2023_2380_MOESM1_ESM.docx]

**SUPPLEMENTARY MATERIAL**

**Supplementary Fig. 1** Trial schema.


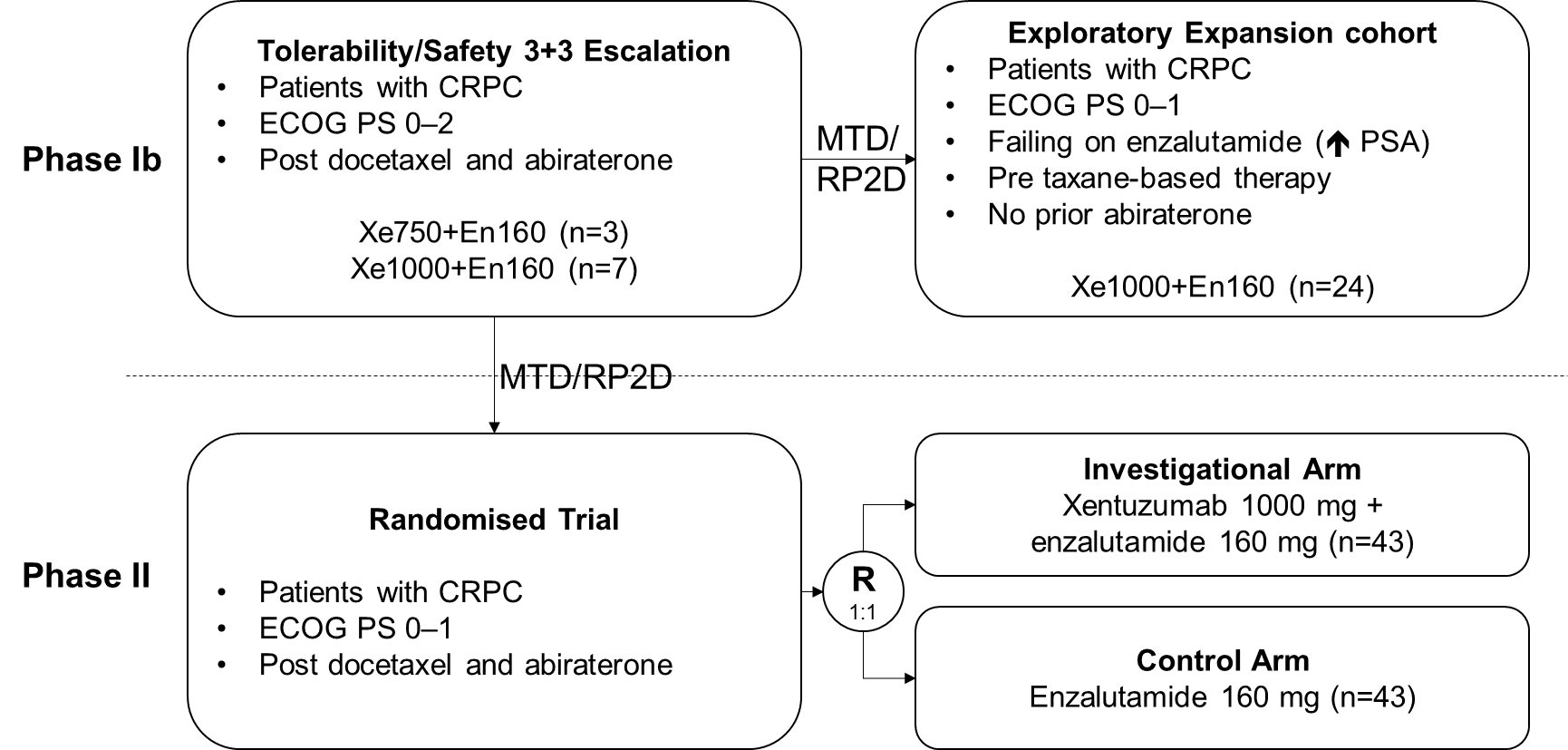


*CRPC* castration-resistant prostate cancer, *ECOG PS* Eastern Cooperative Oncology Group performance status, *En160* enzalutamide 160 mg daily, *MTD* maximum tolerated dose, *PSA* prostate specific antigen, *RP2D* recommended dose for phase II, *Xe750+En160* xentuzumab 750 mg weekly plus enzalutamide 160 mg daily, *Xe1000+En160* xentuzumab 1000 mg weekly plus enzalutamide 160 mg daily.

In both phases, patients received treatment until clinical disease progression or occurrence of undue toxicity or until discontinuation for any other reason.

**Supplementary Table 1.** Most common drug-related adverse events reported in the Phase Ib dose escalation (occurring in ≥2 patients).

|  | **Xe750/1000+En160**  ***N* = 10** | |
| --- | --- | --- |
|  | **All grades** | **Grade 3*** |
| Any DRAE, *n* (%) | 9 (90.0) | 3 (30.0) |
| Fatigue, *n* (%) | 7 (70.0) | 3 (30.0) |
| Decreased appetite, *n* (%) | 5 (50.0) | 0 |
| Nausea, *n* (%) | 3 (30.0) | 0 |
| Weight decreased, *n* (%) | 2 (20.0) | 0 |
| Taste disorder, *n* (%) | 2 (20.0) | 0 |

*DRAE* drug-related adverse event, *Xe750/1000+En160* xentuzumab 750/1000 mg weekly plus enzalutamide 160 mg daily.

*There were no Grade 4 or 5 DRAEs.

**Supplementary Table 2.** Most common drug-related adverse events reported in the Phase Ib dose expansion (occurring in >10% of patients).

|  | **Xe1000+En160**  ***N* = 24** | |
| --- | --- | --- |
|  | **All grades** | **Grade 3*** |
| Any DRAE, *n* (%) | 21 (87.5) | 5 (20.8) |
| Fatigue, *n* (%) | 6 (25.0) | 0 |
| Decreased appetite, *n* (%) | 5 (20.8) | 0 |
| Nausea, *n* (%) | 4 (16.7) | 0 |
| Weight decreased, *n* (%) | 4 (16.7) | 0 |
| Dizziness, *n* (%) | 4 (16.7) | 0 |
| Asthenia, *n* (%) | 3 (12.5) | 1 (4.2) |
| Infusion-related reaction, *n* (%) | 3 (12.5) | 0 |
| Hyperglycaemia, *n* (%) | 3 (12.5) | 0 |
| Muscle spasms | 3 (12.5) | 0 |

*DRAE* drug-related adverse event, *Xe1000+En160* xentuzumab 1000 mg weekly plus enzalutamide 160 mg daily.

*There were no Grade 4 or 5 DRAEs.

**SUPPLEMENTARY METHODS**

Dose-limiting toxicities (DLTs) were defined as any of the following drug-related adverse events (AEs):

- Aspartate aminotransferase (AST) or alanine aminotransferase (ALT) >5 x the upper limit of normal (ULN; for those with baseline AST/ALT ≤ ULN) or AST or ALT > (baseline value + 4x ULN [for those with baseline AST/ALT > ULN])
- Grade 3 or 4 toxicity (except for incompletely treated nausea, untreated vomiting, untreated diarrhoea, fatigue, infusion reaction, electrolyte, or AST/ALT)
- Grade ≥2 infusion reaction despite adequate pre-medication
- Grade ≥2 nausea and/or vomiting persisting for ≥7 days despite antiemetic treatment
- Any grade 4 hyperglycaemia (symptomatic or asymptomatic)
- Any grade 3 hyperglycaemia lasting >48 hours
- Any electrolyte grade 3 AE which was refractory to optimal correction therapy
- Seizure event of any grade
- No recovery from a non-DLT grade >2 toxicity to grade 1 within 14 days of drug administration
- Sustained fatigue/asthenia grade 3 for longer than 96 hours associated with deterioration of Eastern Cooperative Oncology Group performance status
- Any other study drug-related toxicity at any timepoint during the study considered significant enough to be qualified as DLT in the opinion of the investigators and confirmed by the safety review with the sponsor
